# Habitual coffee consumption and genetic predisposition to obesity: gene-diet interaction analyses in three US prospective studies

**DOI:** 10.1186/s12916-017-0862-0

**Published:** 2017-05-09

**Authors:** Tiange Wang, Tao Huang, Jae H. Kang, Yan Zheng, Majken K. Jensen, Janey L. Wiggs, Louis R. Pasquale, Charles S. Fuchs, Hannia Campos, Eric B. Rimm, Walter C. Willett, Frank B. Hu, Lu Qi

**Affiliations:** 1 Department of Epidemiology, School of Public Health and Tropical Medicine, Tulane University, New Orleans, LA 70112 USA; 2 Shanghai Institute of Endocrine and Metabolic Diseases, Rui Jin Hospital, Shanghai Jiao Tong University School of Medicine, Shanghai, China; 3 Channing Division of Network Medicine, Department of Medicine, Brigham and Women’s Hospital and Harvard Medical School, Boston, MA USA; 4 Department of Nutrition, Harvard T.H. Chan School of Public Health, Boston, MA USA; 5 Department of Ophthalmology, Massachusetts Eye and Ear Infirmary, Harvard Medical School, Boston, MA USA; 6 Department of Medical Oncology, Dana-Farber Cancer Institute, Harvard Medical School, Boston, MA USA; 7 Center for Research and Innovation in Translational Nutrition and Health (CIINT), Universidad Hispanoamericana, San Jose, Costa Rica; 8 Department of Epidemiology, Harvard T.H. Chan School of Public Health, Boston, MA USA

**Keywords:** Gene-diet interaction, Coffee, Genetic predisposition, Body mass index, Obesity

## Abstract

**Background:**

Whether habitual coffee consumption interacts with the genetic predisposition to obesity in relation to body mass index (BMI) and obesity is unknown.

**Methods:**

We analyzed the interactions between genetic predisposition and habitual coffee consumption in relation to BMI and obesity risk in 5116 men from the Health Professionals Follow-up Study (HPFS), in 9841 women from the Nurses’ Health Study (NHS), and in 5648 women from the Women’s Health Initiative (WHI). The genetic risk score was calculated based on 77 BMI-associated loci. Coffee consumption was examined prospectively in relation to BMI.

**Results:**

The genetic association with BMI was attenuated among participants with higher consumption of coffee than among those with lower consumption in the HPFS (*P*_*interaction*_ = 0.023) and NHS (*P*_*interaction*_ = 0.039); similar results were replicated in the WHI (*P*_*interaction*_ = 0.044). In the combined data of all cohorts, differences in BMI per increment of 10-risk allele were 1.38 (standard error (SE), 0.28), 1.02 (SE, 0.10), and 0.95 (SE, 0.12) kg/m^2^ for coffee consumption of < 1, 1–3 and > 3 cup(s)/day, respectively (*P*_*interaction*_ < 0.001). Such interaction was partly due to slightly higher BMI with higher coffee consumption among participants at lower genetic risk and slightly lower BMI with higher coffee consumption among those at higher genetic risk. Each increment of 10-risk allele was associated with 78% (95% confidence interval (CI), 59–99%), 48% (95% CI, 36–62%), and 43% (95% CI, 28–59%) increased risk for obesity across these subgroups of coffee consumption (*P*_*interaction*_ = 0.008). From another perspective, differences in BMI per increment of 1 cup/day coffee consumption were 0.02 (SE, 0.09), –0.02 (SE, 0.04), and –0.14 (SE, 0.04) kg/m^2^ across tertiles of the genetic risk score.

**Conclusions:**

Higher coffee consumption might attenuate the genetic associations with BMI and obesity risk, and individuals with greater genetic predisposition to obesity appeared to have lower BMI associated with higher coffee consumption.

**Electronic supplementary material:**

The online version of this article (doi:10.1186/s12916-017-0862-0) contains supplementary material, which is available to authorized users.

## Background

Obesity is a worldwide pandemic that imposes an enormous burden on public health. The rapid rise of obesity prevalence over the past three decades has been primarily attributed to the dramatic changes in diet and lifestyle. As a multifactorial condition, obesity is also determined by genetic makeup. Recently, emerging data suggest that synergistic interactions of genetic predisposition with diet and lifestyle may play an important role in affecting obesity risk [1, 2].

Coffee is among the most widely consumed beverages around the world. The majority of observational studies have associated regular coffee consumption with reduced risk of obesity and related cardiometabolic diseases such as type 2 diabetes [3, 4], but the association between coffee consumption and obesity are not entirely consistent [5–7]. Such heterogeneous associations might be attributed, at least partly, to the modification effects of divergent genetic predispositions, as observed in previous studies showing coffee-gene interactions on other health outcomes [8, 9]. In addition, our previous studies found that the genetic predisposition to obesity might amplify the effects of specific dietary factors on BMI and obesity risk [1, 2]. Therefore, we hypothesized that the association between habitual coffee consumption and reduced adiposity might be amplified by the genetic predisposition to obesity, and that the genetic association with adiposity might also be modified by higher coffee consumption.

To test this hypothesis, we examined the interaction between habitual coffee consumption and a genetic risk score, a marker of the overall genetic predisposition to obesity, in relation to body mass index (BMI) and risk of obesity in US men and women from two prospective cohorts: the Health Professionals Follow-up Study (HPFS) and the Nurses’ Health Study (NHS). We replicated the analyses in an independent, prospective cohort from the Women’s Health Initiative (WHI).

## Methods

### Study population

The HPFS is a prospective cohort study of 51,529 male health professionals aged 40 to 75 years old at the study inception in 1986 [10]. The NHS is a prospective cohort study of 121,700 female registered nurses aged 30 to 55 years old at the study inception in 1976 [11]. In both cohorts, participants were followed using biennial validated questionnaires to update the information on medical history, lifestyle, and health conditions. For the present analyses, we used 1986 as the baseline for the HPFS and NHS, and included 5116 initially healthy men and 9841 initially healthy women of European ancestry for whom baseline decaffeinated and caffeinated coffee consumption and genotype data based on genome-wide association studies (GWAS) were available [12–15]. The institutional review boards of Brigham and Women’s Hospital and Harvard T.H. Chan School of Public Health approved the study protocol.

The WHI is a long-term national health study of 161,808 postmenopausal women aged 50 to 79 years old enrolled between 1993 and 1998 [16, 17]. The datasets used for the analyses were obtained after authorized access from the National Center for Biotechnology Information database of Genotypes and Phenotypes (dbGap; available at: https://www.ncbi.nlm.nih.gov/gap). Phenotypic data were extracted from the WHI Clinical Trial and Observational Study (dbGaP study accession number: phs000200.v10.p3). Genotype data were extracted from the WHI Memory Study + GWAS (phs000675.v2.p3). The current analyses included 5648 initially healthy women of European ancestry with available data at baseline.

### Assessment of coffee consumption

In the HPFS and NHS, dietary information was collected from a validated 131-item semiquantitative food frequency questionnaire (FFQ), administered in 1986 and every 4 years thereafter [18, 19]. Participants were asked how often on average (from “never or less than once per month” to “6 or more times per day”) they consumed a standard portion size of decaffeinated and caffeinated coffee (“one cup”). Total coffee consumption was calculated as the sum of decaffeinated and caffeinated coffee consumption. The FFQ assessment of coffee consumption was validated to be highly correlated with the diet record assessment (r = 0.78) [20].

In the WHI, dietary intake was measured by using a validated 145-item semiquantitative FFQ, administered in 1993 and every year thereafter [21]. Women reported coffee consumption according to medium serving, which was defined as one standard portion size of coffee and was equivalent to “one cup”. The FFQ did not differentiate between caffeinated and decaffeinated coffee.

### Assessment of BMI and covariates

In the HPFS and NHS, height and body weight were assessed by a questionnaire at baseline, and weight was requested on each follow-up questionnaire. Self-reported weights were highly correlated with measured weights (r = 0.97 for women in the NHS and 0.97 for men in the HPFS) in a validation subsample [22]. BMI was calculated as weight in kilograms divided by the square of height in meters. Obesity is defined as a BMI of 30 kg/m^2^ or higher. Information on demographics, medical history, and lifestyle behaviors was derived from the biennial questionnaires. Physical activity was expressed as metabolic equivalents per week calculated using the reported time spent on various activities, weighting each activity by its intensity level. The validation of physical activity has been described previously [23]. Dietary variables (alcohol and sugar-sweetened beverages) were assessed by validated FFQs [18, 19]. Sugar-sweetened beverages included caffeinated colas, caffeine-free colas, carbonated non-cola soft drinks, and noncarbonated sugar-sweetened beverages [1]. Diet quality was assessed by using the Alternative Healthy Eating Index, with a higher score indicating a healthier diet [24].

In the WHI, height was measured with a stadiometer and weight was measured while the participants were wearing light clothing. Information on demographics, medical history, and lifestyle behaviors was collected by the standardized questionnaires. Dietary variables (alcohol, sugar-sweetened beverages, and the Alternative Healthy Eating Index) were derived or calculated from participant responses to the FFQs [21].

### Genotyping and calculation of genetic risk score

We selected 77 single nucleotide polymorphisms (SNPs) that represent all 77 loci associated with BMI at a genome-wide significance level (Additional file 1: Table S1) in European individuals [25]. In the HPFS and NHS, SNP genotyping and imputation have been described in detail elsewhere [12–15]. We used MACH (http://www.sph.umich.edu/csg/abecasis/mach) to impute SNPs on chromosomes 1–22, with National Center for Biotechnology Information build 36 of phase II HapMap CEU data (release 22) as the reference panel. Most of the SNPs were genotyped or had a high imputation quality score (MACH r^2^ ≥ 0.8). In the WHI, all 77 SNPs were successfully extracted.

The genetic risk score was calculated on the basis of the 77 SNPs by using a weighted method according to each SNP’s relative effect size (β coefficient) obtained from the GWAS [25]. We rescaled the weighted score to reflect the number of risk alleles – each point of the genetic risk score corresponded to one risk allele. The genetic risk score ranges from 0 to 154, with higher scores indicating a greater genetic predisposition to obesity.

### Statistical analysis

In the HPFS and NHS, we analyzed the data prospectively with the assessment of coffee consumption 4 years before the assessment of BMI and obesity in order to minimize reverse causality. We used follow-up data up to 1998 because the trend of adult obesity prevalence in the US had a marked increase during 1985 to 1999 and then became stable [26], making the period before 1999 ideal for studying a gene-environment interaction on obesity and also for minimizing confounding from the loss of lean muscle mass at older ages since the mean age of the participants in the two cohorts was over 65 years after 1998 (age range, 51–85 years in the HPFS and 51–77 years in the NHS) [2]. Generalized linear models with repeated-measures analyses were used to estimate the differences in BMI per increment of 10-risk allele, stratified according to subgroups of coffee consumption. Odds ratios for prevalent obesity for each increment of 10-risk allele were transformed from β coefficients calculated by generalized linear models. We also examined the differences in BMI per increment of 1 cup/day coffee consumption according to tertiles of the genetic risk score. The effects of interactions between the genetic risk score and coffee consumption on BMI or prevalent obesity were tested by including the respective multiplicative interaction terms in the models. As for sensitivity analysis, we analyzed the interaction between coffee consumption and the genetic risk score on BMI and obesity in participants excluding non-coffee consumers or in never smokers to assess whether the interaction effect may be influenced by non-coffee consumers or smoking status. We further examined the interactions for decaffeinated and caffeinated coffee in the secondary analyses. The analyses were repeated in the WHI to replicate the findings in the HPFS and NHS. In the WHI, data were prospectively analyzed with the assessment of coffee consumption before the assessment of BMI and obesity, and two repeated measures were analyzed during 1993 to 2003. In three cohorts, except for diet and BMI, missing data during any follow-up period were coded as a missing indicator category for categorical variables (e.g., smoking status) or with carried-forward values for continuous variables. Missing values for diet and BMI were carried forward only once and after that the follow-up was censored. Results across studies were pooled with inverse-variance-weighted meta-analyses by random effects models (if *P* < 0.05 for heterogeneity) or fixed effects models (if *P* ≥ 0.05 for heterogeneity). All reported *P* values are nominal and two-sided. Statistical analyses were performed in SAS 9.4 (SAS Institute, Cary, NC, USA).

## Results

### Baseline characteristics

The baseline characteristics of the participants from the HPFS, NHS, and WHI are presented in Table 1. The baseline means (SD) consumption of total coffee were 2.09 (1.86), 2.43 (1.83), and 2.42 (1.84) cups/day in HPFS (in 1986), NHS (in 1986), and WHI (in 1993), respectively. Generally, participants who drank coffee more frequently were younger and more likely to be current smokers, and had higher intake of total energy and alcohol. The mean values (SD) of the genetic risk score were 69.3 (5.6) in the HPFS, 69.5 (5.5) in the NHS, and 70.6 (5.6) in the WHI. In all cohorts, the genetic risk score showed similar normal distributions, and was positively associated with BMI (*P* < 0.001 for all cohorts; Additional file 1: Figure S1).

**Table 1:** Baseline characteristics of 20,605 participants in the HPFS, NHS, and WHI, according to coffee consumption

Characteristic	Coffee consumption		
	< 1 cup/day	1–3 cups/day	> 3 cups/day
HPFS^a^			
Participants, n	2141	1787	1188
Age, year	54.6 ± 9.1	54.8 ± 8.5	53.8 ± 8.0
BMI, kg/m^2^	25.1 ± 4.9	25.4 ± 4.6	25.8 ± 4.4
Coffee, cups/day	0.40 ± 0.42	2.26 ± 0.55	4.89 ± 1.14
Physical activity, MET-h/wk	20.2 ± 26.5	19.6 ± 23.1	18.1 ± 23.4
AHEI score	53.1 ± 11.9	52.8 ± 11.6	51.5 ± 11.9
Total energy intake, kcal/day	1994 ± 603	2040 ± 616	2114 ± 638
Alcohol, g/day	9.7 ± 14.7	14.4 ± 16.0	14.8 ± 18.5
Current smokers, %	4.6	8.1	16.6
Sugar-sweetened beverages, serving/day	1.29 ± 1.19	1.16 ± 1.00	0.98 ± 0.90
Genetic risk score	69.3 ± 5.6	69.1 ± 5.5	69.7 ± 5.6
NHS^a^			
Participants, n	2679	4296	2866
Age, year	53.8 ± 7.0	54.4 ± 6.5	53.8 ± 6.4
BMI, kg/m^2^	26.3 ± 5.5	25.7 ± 4.9	25.4 ± 4.6
Coffee, cups/day	0.42 ± 0.42	2.23 ± 0.53	4.60 ± 1.09
Physical activity, MET-h/wk	14.0 ± 17.5	14.5 ± 19.7	13.2 ± 16.9
AHEI score	48.8 ± 10.9	49.8 ± 10.3	49.6 ± 10.3
Total energy intake, kcal/day	1735 ± 512	1762 ± 484	1813 ± 506
Alcohol, g/day	4.5 ± 9.3	7.6 ± 11.0	7.7 ± 11.2
Current smokers, %	9.1	15.2	28.4
Sugar-sweetened beverages, serving/day	1.11 ± 0.96	1.02 ± 0.86	0.85 ± 0.78
Genetic risk score	69.3 ± 5.6	69.5 ± 5.5	69.7 ± 5.5
WHI^a^			
Participants, n	951	3223	1474
Age, year	68.4 ± 5.9	68.4 ± 5.7	67.2 ± 6.1
BMI, kg/m^2^	28.9 ± 5.8	28.1 ± 5.4	28.4 ± 5.6
Coffee, cups/day	0.32 ± 0.27	1.89 ± 0.60	4.94 ± 1.51
Physical activity, MET-h/wk	11.9 ± 13.7	11.7 ± 13.1	10.9 ± 12.4
AHEI score	55.7 ± 9.5	56.8 ± 9.8	54.8 ± 10.1
Total energy intake, kcal/day	1413 ± 640	1570 ± 588	1795 ± 747
Alcohol, g/day	3.4 ± 10.4	6.3 ± 11.3	6.9 ± 14.1
Current smokers, %	4.5	6.6	10.9
Sugar-sweetened beverages, serving/day	0.93 ± 0.98	1.01 ± 1.02	1.04 ± 1.44
Genetic risk score	70.5 ± 5.7	70.4 ± 5.6	71.0 ± 5.4

Plus-minus values are means ± SD

^a^Baseline data were from 5116 men in the HPFS (1986), 9841 women in the NHS (1986), and 5648 women in the WHI (1993)

*AHEI* Alternative Healthy Eating Index, *BMI* body mass index, *MET-h/wk* metabolic equivalent of task hours per week

### Genetic association with BMI by subgroups of coffee consumption

In the HPFS and NHS, the genetic association with BMI was attenuated among participants with higher consumption of coffee than among those with lower consumption (Table 2). After multivariable adjustment, differences in BMI per 10-risk allele increment were 0.81 (SE, 0.25), 0.81 (SE, 0.25), and 0.32 (SE, 0.30) kg/m^2^ for coffee consumption of < 1, 1–3 and > 3 cup(s)/day, respectively, in the HPFS (*P* for interaction = 0.023), and were 1.59 (SE, 0.18), 1.07 (SE, 0.13), and 1.13 (SE, 0.16) kg/m^2^ for the subgroups of coffee consumption, respectively, in the NHS (*P* for interaction = 0.039). Significant interactions were replicated in the WHI (*P* for interaction = 0.044) – compared with participants who consumed less than 1 cup/day of coffee, those who consumed more than 3 cups/day exhibited lower BMI per 10-risk allele increment (0.96 (SE, 0.25) vs. 1.74 (SE, 0.30) kg/m^2^). In the combined data of the three cohorts, differences in BMI per 10-risk allele increment were 1.38 (SE, 0.28) kg/m^2^ for coffee consumption of < 1 cup/day, 1.02 (SE, 0.10) kg/m^2^ for 1–3 cups/day, and 0.95 (SE, 0.12) kg/m^2^ for > 3 cups/day (*P* for interaction < 0.001). In sensitivity analysis, after excluding non-coffee consumers, the interaction pattern was not substantially changed and the interaction effect remained statistically significant (for main baseline characteristics of non-coffee consumers see Additional file 1: Table S2; for results of sensitivity analysis see Additional file 1: Table S3). Further, in never smokers, the interaction effect was attenuated due to smaller sample sizes but remained statistically significant in the combined cohorts (Additional file 1: Table S4).

**Table 2:** Differences in BMI per increment of 10-risk alleles, according to coffee consumption in the HPFS, NHS, and WHI

Analysis	Coffee consumption			*P* for interaction
	< 1 cup/day	1–3 cups/day	> 3 cups/day	
HPFS				
Model 1	0.85 ± 0.25	0.71 ± 0.24	0.29 ± 0.30	0.012
Model 2	0.81 ± 0.25	0.81 ± 0.25	0.32 ± 0.30	0.023
NHS				
Model 1	1.68 ± 0.18	1.13 ± 0.13	1.21 ± 0.16	0.048
Model 2	1.59 ± 0.18	1.07 ± 0.13	1.13 ± 0.16	0.039
WHI				
Model 1	1.71 ± 0.30	1.08 ± 0.17	0.97 ± 0.25	0.049
Model 2	1.74 ± 0.30	1.02 ± 0.17	0.96 ± 0.25	0.044
Pooled^a^				
Model 1	1.42 ± 0.28	1.05 ± 0.10	0.87 ± 0.26	<0.001
Model 2	1.38 ± 0.28	1.02 ± 0.10	0.95 ± 0.12	<0.001

Plus-minus values are β coefficients ± SE

In the HPFS and NHS, data were derived from the repeated-measures analysis in men (three measures from 1986 to 1998) and women (three measures from 1986 to 1998); in the WHI, data were derived from the repeated-measures in women (two measures from 1993 to 2003)

Model 1: adjusted for age and genotyping source

Model 2: based on Model 1, further adjusted for physical activity (< 3, 3–8.9, 9–17.9, 18–26.9, ≥ 27 MET-h/wk), Alternative Healthy Eating Index score (quintiles), total energy intake (quintiles), smoking status (never, former, current), sugar-sweetened beverage consumption (quintiles), and alcohol consumption (0, 0.1–4.9, 5–9.9, 10–14.9, ≥ 15 g/day)

^a^Results for the three cohorts were pooled by means of inverse-variance-weighted random effects meta-analysis (if *P* < 0.05 for heterogeneity) or fixed effects meta-analysis (if *P* ≥ 0.05 for heterogeneity)

We also observed similar interaction patterns for decaffeinated and caffeinated coffee in the HPFS and NHS (for baseline characteristics for decaffeinated and caffeinated coffee consumers see Additional file 1: Table S5). In the combined cohorts, differences in BMI per 10-risk allele increment were 1.17 (SE, 0.09), 0.82 (SE, 0.18), and 0.68 (SE, 0.35) kg/m^2^ across the three subgroups of decaffeinated consumption (*P* for interaction = 0.010), and were 1.16 (SE, 0.11), 1.02 (SE, 0.13), and 0.94 (SE, 0.19) kg/m^2^ across the three subgroups of caffeinated consumption (*P* for interaction = 0.212) (Additional file 1: Table S6).

### Genetic association with obesity by subgroups of coffee consumption

**Fig. 1:**
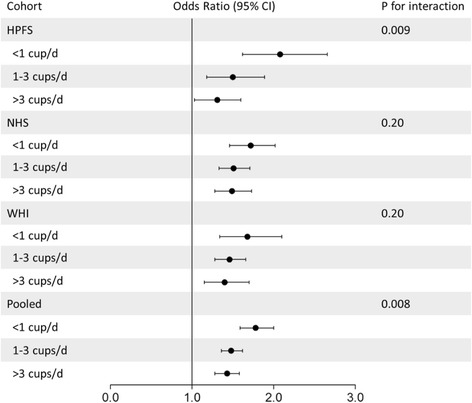
Genetic associations with prevalent obesity, according to coffee consumption in the HPFS, NHS, and WHI. Data are odds ratios (95% CIs) for prevalent obesity. In the HPFS and NHS, data were derived from the repeated-measures analysis in men (three measures from 1986 to 1998) and women (three measures from 1986 to 1998); in the WHI, data were derived from the repeated-measures in women (two measures from 1993 to 2003). Data were adjusted for age, genotyping source, physical activity (<3, 3–8.9, 9–17.9, 18–26.9, ≥ 27 MET-h/wk), Alternative Healthy Eating Index score (quintiles), total energy intake (quintiles), smoking status (never, former, current), sugar-sweetened beverage consumption (quintiles), and alcohol consumption (0, 0.1–4.9, 5–9.9, 10–14.9, ≥ 15 g/day). Results for the three cohorts were pooled by means of inverse-variance-weighted random effects meta-analysis (if *P* < 0.05 for heterogeneity) or fixed effects meta-analysis (if *P* ≥ 0.05 for heterogeneity)

The genetic association with prevalent obesity was attenuated with higher consumption of coffee in the HPFS, NHS, and WHI (Fig. 1). In the HPFS, the multivariable-adjusted odds ratios (95% CI) for obesity per increment of 10-risk allele were 2.08 (1.62–2.66) for coffee consumption of < 1 cup/day, 1.50 (1.18–1.89) for 1–3 cups/day, and 1.31 (1.03–1.67) for > 3 cups/day (*P* for interaction = 0.009). Similar interactions were also observed in the NHS and WHI. In the combined cohorts, each 10-risk allele increment was associated with 78% (95% CI, 59–99%), 48% (95% CI, 36–62%), and 43% (95% CI, 28–59%) increased risk for obesity across these three subgroups of coffee consumption (*P* for interaction = 0.008). In participants excluding non-coffee consumers, or in never smokers, the interaction pattern was not substantially changed (Additional file 1: Table S3 and Table S4).

### Association between coffee consumption and BMI by genetic risk score in tertiles

**Fig. 2:**
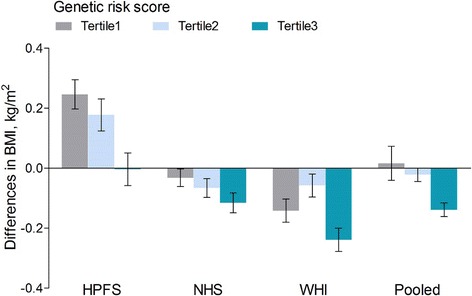
Differences in BMI per increment of 1 cup/day coffee consumption, according to tertiles of the genetic risk score in the HPFS, NHS, and WHI. Data are multivariable-adjusted β coefficients (SEs) of BMI (kg/m^2^). Data description and adjustment are the same as shown in Fig. 1

Differences in BMI associated with coffee consumption gradually decreased with the increased genetic risk in the HPFS, NHS, and WHI (Fig. 2). In the combined cohorts, differences in BMI per increment of 1 cup/day coffee consumption were 0.02 (SE, 0.09), –0.02 (SE, 0.04), and –0.14 (SE, 0.04) kg/m^2^ across tertiles of the genetic risk score. Similar interaction patterns were also observed for decaffeinated and caffeinated coffee: across tertiles of the genetic risk score, each 1 cup/day of decaffeinated coffee consumption was associated with 0.08 (SE, 0.06), 0.03 (SE, 0.06), and –0.15 (SE, 0.07) kg/m^2^ differences in BMI, and each 1 cup/day of caffeinated coffee consumption was associated with 0.03 (SE, 0.05), 0.00 (SE, 0.05), and –0.06 (SE, 0.05) kg/m^2^ differences in BMI (Additional file 1: Figure S2).

### BMI according to joint categories of coffee consumption and the genetic risk score

**Fig. 3:**
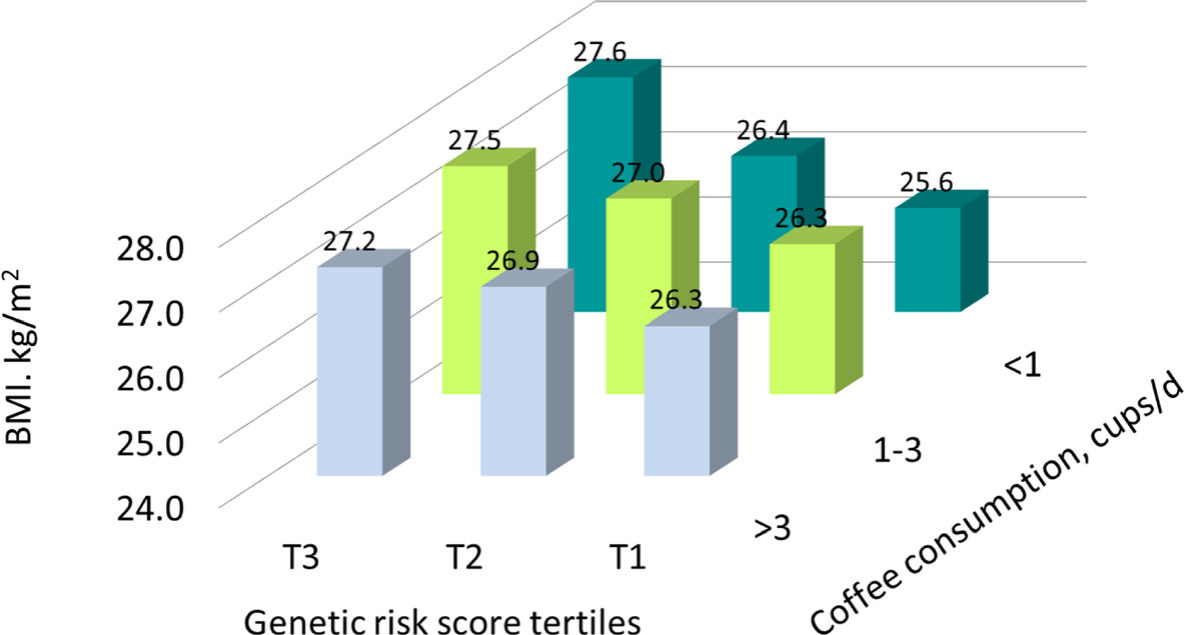
BMI according to joint categories of coffee consumption and the genetic risk score in combined data of the HPFS, NHS, and WHI. Data are multivariable-adjusted mean values of BMI (kg/m^2^). Data description and adjustment are the same as shown in Fig. 1

In the combined data of the three cohorts, higher genetic risk score was associated with higher BMI, and such an association was more prominent among participants who habitually consumed more than 1 cup/day of coffee than among those who consumed coffee more frequently. Viewed differently, BMI levels were slightly higher with higher coffee consumption among participants at lower genetic risk, and were slightly lower with higher coffee consumption among those at higher genetic risk (Fig. 3).

## Discussion

In three prospective cohorts of US men and women, we, for the first time, found consistently significant interactions between habitual coffee consumption and genetic predisposition in relation to BMI and obesity. In all three cohorts, the combined genetic effects on BMI and risk of obesity among individuals consuming one or more cups of coffee were approximately 30% lower than among those consuming less than one cup of coffee. These findings suggest that higher coffee consumption might attenuate the genetic influences on adiposity. From another perspective, individuals with greater genetic predisposition to obesity appeared to have lower BMI associated with higher coffee consumption, suggesting that the association of coffee consumption with reduced adiposity might be strengthened by the genetic predisposition. Such findings were in line with our hypothesis.

Multiple epidemiological studies have shown inverse associations of habitual coffee consumption with obesity, weight gain, and related cardiometabolic diseases [3, 4, 27]. However, a handful of studies also highlighted that individuals with higher coffee consumption might have higher BMI [5–7]. In this study, we provided novel evidence from an aspect of gene-diet interaction that habitual coffee consumption might lower BMI and obesity risk by interacting with the genetic predisposition to obesity. In general, higher coffee consumption seemed to be associated with higher BMI in the HPFS but lower BMI in the NHS and WHI, which may be partially due to possible sex-specific health effects of coffee as noted by several studies [28, 29]. Although the differences in BMI across coffee consumption levels appeared to be slight and the associations were inconsistent among men and women, we observed consistent results across all cohorts that individuals with higher genetic risk to obesity tended to have lower BMI associated with higher coffee consumption. The significant interaction effect may be partly due to the positive association between coffee consumption and BMI among individuals at low genetic risk and the inverse association among those at high genetic risk. These results were in line with our previous findings that individuals with divergent genetic predispositions might be differently related to adiposity according to certain dietary factors [1, 2], and might partly explain the heterogeneous associations between coffee consumption and adiposity reported by previous studies [3–7, 27]. Viewed differently, the higher BMI or obesity risk attributed to genetic predisposition appeared to be attenuated in individuals who consumed coffee more frequently. Additionally, the interactions between coffee consumption and the genetic risk score on BMI and obesity remained stable in participants excluding non-coffee consumers or in never smokers, which suggest that the interactions were less likely to be largely influenced by non-coffee consumers or smoking status. Taken together, our findings lend support to the evidence that habitual coffee consumption and genetic predisposition may interact with each other and synergistically influence adiposity.

The mechanisms involved in the interaction between coffee consumption and the genetic predisposition to obesity are yet unclear. Coffee contains several biologically active compounds that have beneficial effects on obesity and related cardiometabolic diseases, e.g., chlorogenic acid, caffeine, trigonelline, and magnesium are most likely to accelerate weight loss through their antioxidant, hypoglycemic, and hypolipidemic functions [30–33]. In addition, a number of BMI-associated genes are highly expressed in the brain and hypothalamus, and play essential roles through the central nervous system in appetite control, food preference, and energy homeostasis [25]. Future functional experiments are needed to explore the pathways underlying such gene-diet interactions that lead to obesity.

The strengths of this study include the prospectively designed large cohorts with well-validated information on dietary and lifestyle factors, the genetic risk score integrating comprehensive genetic information of recently identified genetic variants, the use of repeated measures of coffee consumption and BMI, and the consistent findings across all three cohorts. Nevertheless, several limitations should be taken into consideration. First, although we have carefully controlled for multiple diet and lifestyle factors, residual confounding by other unmeasured or unknown factors could not be fully eliminated. Second, in the HPFS and NHS, the data on weight and height are self-reported, and measurement errors in diet and lifestyle factors are inevitable. However, validation studies found that the FFQs and the self-reported weight and height were highly reliable in these cohorts [18–23]. Third, the genetic risk score integrated genetic information from all established genetic variants associated with BMI, but these genetic loci account for only a small amount of BMI variation [25]. Fourth, we observed similar gene-coffee interactions for decaffeinated and caffeinated coffee, but only the interaction for decaffeinated coffee reached statistical significance. Although we have adjusted for several confounders (decaffeinated and caffeinated coffee were adjusted for each other) in the analyses, residual confounding might still exist, and the exact mechanism underlying the observed difference was not clear. Fifth, the analyses of this study were restricted to Caucasian participants and those with genotyping data (comparison results of the baseline characteristics for the study participants and the other participants are shown in Additional file 1: Table S7); thus, the study participants could not represent the full cohorts and it is unknown whether the findings can be generalized to other ethnic groups.

## Conclusions

In conclusion, our data provide consistent evidence that habitual coffee consumption might attenuate the genetic influences on higher BMI and obesity risk among US men and women, and the association of coffee consumption with reduced adiposity was more prominent in individuals with greater genetic predisposition.

## Additional file

Additional file 1: Table S1. Characteristics of 77 SNPs for BMI in HPFS, NHS, and WHI. **Table S2.** Baseline characteristics of non-coffee consumers in HPFS, NHS, and WHI. **Table S3.** Genetic associations with differences in BMI and prevalent obesity, according to coffee consumption in HPFS, NHS, and WHI (excluding non-coffee consumers). **Table S4.** Genetic associations with differences in BMI and prevalent obesity, according to coffee consumption in HPFS, NHS and WHI (only in non-smokers). **Table S5.** Baseline characteristics of decaffeinated coffee consumers and caffeinated coffee consumers in HPFS and NHS. **Table S6.** Differences in BMI per increment of 10-risk alleles, according to consumption of decaffeinated coffee and caffeinated coffee in HPFS and NHS. **Table S7.** Comparison of baseline characteristics for study participants and the other participants in the HPFS and NHS. **Figure S1**. Distribution of the genetic risk score in the HPFS, NHS, and WHI. **Figure S2**. Differences in BMI per increment of 1 cup/day decaffeinated coffee and caffeinated coffee consumption, according to tertiles of the genetic risk score in HPFS and NHS. (DOCX 687 kb)

## Abbreviations

BMI: Body mass index
FFQ: Food frequency questionnaire
GWAS: Genome-wide association study
HPFS: Health Professionals Follow-up Study
NHS: Nurses’ Health Study
SNP: Single nucleotide polymorphism
WHI: Women’s Health Initiative
