# Supplementary material for: Habitual coffee consumption and genetic predisposition to obesity: gene-diet interaction analyses in three US prospective studies
Source: BMC Med. 2017 May 9;15:97. doi: 10.1186/s12916-017-0862-0 (PMC5424298; doi:10.1186/s12916-017-0862-0)

**Habitual coffee consumption and genetic predisposition to obesity: gene-diet interaction analyses in three US prospective studies**

Supplementary Material

Contents

| Table S1 | Characteristics of 77 SNPs for BMI in the HPFS, NHS and WHI | 2 |
| --- | --- | --- |
| Table S2 | Baseline characteristics of non-coffee consumers in the HPFS, NHS and WHI | 4 |
| Table S3 | Genetic associations with differences in BMI and prevalent obesity, according to coffee consumption in the HPFS, NHS and WHI (excluding non-coffee consumers) | 5 |
| Table S4 | Genetic associations with differences in BMI and prevalent obesity, according to coffee consumption in the HPFS, NHS and WHI (only in non-smokers) | 6 |
| Table S5 | Baseline characteristics of decaffeinated coffee consumers and caffeinated coffee consumers in the HPFS and NHS | 7 |
| Table S6 | Differences in BMI per increment of 10 risk alleles, according to consumptions of decaffeinated coffee and caffeinated coffee in the HPFS and NHS | 8 |
| Table S7 | Comparison of baseline characteristics for study participants and the other participants in the HPFS and NHS | 9 |
| Figure S1 | Distribution of the genetic risk score in the HPFS, NHS and WHI | 10 |
| Figure S2 | Differences in BMI per increment of 1 cup/d decaffeinated coffee and caffeinated coffee consumption, according to tertiles of the genetic risk score in the HPFS and NHS | 11 |

**Table S1. Characteristics of 77 SNPs for BMI in the HPFS, NHS and WHI**

| SNPs | Nearest gene | Chr. | Alleles |  | Frequency of Effect allele, % | | |
| --- | --- | --- | --- | --- | --- | --- | --- |
|  |  |  | Effect/other |  | HPFS | NHS | WHI |
| rs657452 | *AGBL4* | 1 | A/G |  | 0.37 | 0.38 | 0.40 |
| rs2820292 | *NAV1* | 1 | C/A |  | 0.52 | 0.53 | 0.55 |
| rs11583200 | *ELAVL4* | 1 | C/T |  | 0.37 | 0.39 | 0.39 |
| rs543874 | *SEC16B* | 1 | G/A |  | 0.19 | 0.19 | 0.19 |
| rs3101336 | *NEGR1* | 1 | C/T |  | 0.64 | 0.63 | 0.62 |
| rs12566985 | *FPGT-TNNI3K* | 1 | G/A |  | 0.43 | 0.43 | 0.44 |
| rs17024393 | *GNAT2* | 1 | C/T |  | 0.03 | 0.03 | 0.03 |
| rs11165643 | *PTBP2* | 1 | T/C |  | 0.58 | 0.59 | 0.58 |
| rs12401738 | *FUBP1* | 1 | A/G |  | 0.32 | 0.34 | 0.35 |
| rs7599312 | *ERBB4* | 2 | G/A |  | 0.74 | 0.73 | 0.74 |
| rs11126666 | *KCNK3* | 2 | A/G |  | 0.26 | 0.27 | 0.27 |
| rs1528435 | *UBE2E3* | 2 | T/C |  | 0.62 | 0.63 | 0.62 |
| rs11688816 | *EHBP1* | 2 | G/A |  | 0.48 | 0.51 | 0.52 |
| rs13021737 | *TMEM18* | 2 | G/A |  | 0.81 | 0.82 | 0.82 |
| rs10182181 | *ADCY3* | 2 | G/A |  | 0.47 | 0.47 | 0.47 |
| rs1016287 | *FLJ30838* | 2 | T/C |  | 0.30 | 0.29 | 0.30 |
| rs2121279 | *LRP1B* | 2 | T/C |  | 0.14 | 0.13 | 0.13 |
| rs2365389 | *FHIT* | 3 | C/T |  | 0.60 | 0.59 | 0.59 |
| rs16851483 | *RASA2* | 3 | T/G |  | 0.07 | 0.06 | 0.06 |
| rs6804842 | *RARB* | 3 | G/A |  | 0.59 | 0.57 | 0.58 |
| rs3849570 | *GBE1* | 3 | A/C |  | 0.32 | 0.34 | 0.33 |
| rs1516725 | *ETV5* | 3 | C/T |  | 0.86 | 0.87 | 0.86 |
| rs13078960 | *CADM2* | 3 | G/T |  | 0.22 | 0.20 | 0.21 |
| rs17001654 | *SCARB2* | 4 | G/C |  | 0.17 | 0.16 | 0.16 |
| rs11727676 | *HHIP* | 4 | T/C |  | 0.91 | 0.92 | 0.90 |
| rs10938397 | *GNPDA2* | 4 | G/A |  | 0.44 | 0.44 | 0.43 |
| rs13107325 | *SLC39A8* | 4 | T/C |  | 0.08 | 0.07 | 0.07 |
| rs2112347 | *POC5* | 5 | T/G |  | 0.63 | 0.64 | 0.64 |
| rs13191362 | *PARK2* | 6 | A/G |  | 0.88 | 0.88 | 0.88 |
| rs2033529 | *TDRG1* | 6 | G/A |  | 0.29 | 0.28 | 0.29 |
| rs9400239 | *FOXO3* | 6 | C/T |  | 0.70 | 0.70 | 0.70 |
| rs2207139 | *TFAP2B* | 6 | G/A |  | 0.17 | 0.17 | 0.17 |
| rs205262 | *C6orf106* | 6 | G/A |  | 0.28 | 0.27 | 0.28 |
| rs1167827 | *HIP1* | 7 | G/A |  | 0.57 | 0.57 | 0.58 |
| rs2245368 | *PMS2L11* | 7 | C/T |  | 0.16 | 0.16 | 0.18 |
| rs2033732 | *RALYL* | 8 | C/T |  | 0.75 | 0.75 | 0.75 |
| rs17405819 | *HNF4G* | 8 | T/C |  | 0.70 | 0.70 | 0.69 |
| rs1928295 | *TLR4* | 9 | T/C |  | 0.54 | 0.56 | 0.55 |
| rs4740619 | *C9orf93* | 9 | T/C |  | 0.46 | 0.44 | 0.45 |
| rs10733682 | *LMX1B* | 9 | A/G |  | 0.49 | 0.49 | 0.48 |
| rs6477694 | *EPB41L4B* | 9 | C/T |  | 0.33 | 0.34 | 0.34 |
| rs10968576 | *LINGO2* | 9 | G/A |  | 0.31 | 0.31 | 0.31 |
| rs7903146 | *TCF7L2* | 10 | C/T |  | 0.70 | 0.70 | 0.71 |
| rs17094222 | *HIF1AN* | 10 | C/T |  | 0.22 | 0.21 | 0.22 |
| rs11191560 | *NT5C2* | 10 | C/T |  | 0.09 | 0.09 | 0.09 |
| rs7899106 | *GRID1* | 10 | G/A |  | 0.05 | 0.05 | 0.05 |
| rs12286929 | *CADM1* | 11 | G/A |  | 0.47 | 0.47 | 0.46 |
| rs2176598 | *HSD17B12* | 11 | T/C |  | 0.25 | 0.25 | 0.25 |
| rs11030104 | *BDNF* | 11 | A/G |  | 0.78 | 0.79 | 0.79 |
| rs3817334 | *MTCH2* | 11 | T/C |  | 0.41 | 0.41 | 0.41 |
| rs4256980 | *TRIM66* | 11 | G/C |  | 0.63 | 0.64 | 0.64 |
| rs11057405 | *CLIP1* | 12 | G/A |  | 0.92 | 0.90 | 0.91 |
| rs7138803 | *BCDIN3D* | 12 | A/G |  | 0.40 | 0.38 | 0.38 |
| rs12429545 | *OLFM4* | 13 | A/G |  | 0.13 | 0.13 | 0.12 |
| rs12016871 | *MTIF3* | 13 | T/C |  | 0.18 | 0.19 | 0.09 |
| rs10132280 | *STXBP6* | 14 | C/A |  | 0.67 | 0.69 | 0.69 |
| rs12885454 | *PRKD1* | 14 | C/A |  | 0.59 | 0.66 | 0.66 |
| rs7141420 | *NRXN3* | 14 | T/C |  | 0.48 | 0.53 | 0.53 |
| rs11847697 | *PRKD1* | 14 | T/C |  | 0.04 | 0.05 | 0.05 |
| rs3736485 | *DMXL2* | 15 | A/G |  | 0.45 | 0.46 | 0.46 |
| rs16951275 | *MAP2K5* | 15 | T/C |  | 0.75 | 0.77 | 0.76 |
| rs758747 | *NLRC3* | 16 | T/C |  | 0.29 | 0.27 | 0.29 |
| rs9925964 | *KAT8* | 16 | A/G |  | 0.61 | 0.63 | 0.63 |
| rs2650492 | *SBK1* | 16 | A/G |  | 0.32 | 0.32 | 0.25 |
| rs1558902 | *FTO* | 16 | A/T |  | 0.43 | 0.42 | 0.41 |
| rs3888190 | *ATP2A1* | 16 | A/C |  | 0.37 | 0.39 | 0.39 |
| rs12446632 | *GPRC5B* | 16 | G/A |  | 0.86 | 0.86 | 0.86 |
| rs1000940 | *RABEP1* | 17 | G/A |  | 0.28 | 0.28 | 0.31 |
| rs12940622 | *RPTOR* | 17 | G/A |  | 0.58 | 0.57 | 0.57 |
| rs7243357 | *GRP* | 18 | T/G |  | 0.83 | 0.83 | 0.82 |
| rs6567160 | *MC4R* | 18 | C/T |  | 0.23 | 0.24 | 0.23 |
| rs1808579 | *C18orf8* | 18 | C/T |  | 0.44 | 0.46 | 0.48 |
| rs17724992 | *PGPEP1* | 19 | A/G |  | 0.73 | 0.74 | 0.73 |
| rs2287019 | *QPCTL* | 19 | C/T |  | 0.82 | 0.82 | 0.80 |
| rs3810291 | *ZC3H4* | 19 | A/G |  | 0.33 | 0.32 | 0.32 |
| rs2075650 | *TOMM40* | 19 | A/G |  | 0.90 | 0.88 | 0.87 |
| rs29941 | *KCTD15* | 19 | G/A |  | 0.68 | 0.68 | 0.67 |

Chr., chromosome.

Allele coding based on the forward strand. Effect allele is the one associated with high BMI; and other is the reference allele.

**Table S2. Baseline characteristics of non-coffee consumers in the HPFS, NHS and WHI**

| Characteristic | Non-coffee consumers |
| --- | --- |
| **HPFS** |  |
| Participants, n | 794 |
| Age, year | 53.1 ± 9.0 |
| BMI, kg/m^2^ | 25.0 ± 4.4 |
| Coffee, cups/d | 0 |
| Physical activity, MET-h/wk | 19.9 ± 25.1 |
| AHEI score | 51.8 ± 11.8 |
| Total energy intake, kcal/d | 2003 ± 622 |
| Alcohol, g/d | 6.5 ± 12.7 |
| Current smokers, % | 3.8 |
| Sugar-sweetened beverages, serving/d | 1.41 ± 1.38 |
| Genetic risk score | 68.8 ± 5.5 |
| **NHS** |  |
| Participants, n | 907 |
| Age, year | 53.2 ± 7.1 |
| BMI, kg/m^2^ | 26.8 ± 5.7 |
| Coffee, cups/d | 0 |
| Physical activity, MET-h/wk | 13.8 ± 17.2 |
| AHEI score | 47.4 ± 11.2 |
| Total energy intake, kcal/d | 1736 ± 537 |
| Alcohol, g/d | 3.1 ± 8.4 |
| Current smokers, % | 10.3 |
| Sugar-sweetened beverages, serving/d | 1.14 ± 1.09 |
| Genetic risk score | 69.3 ± 5.6 |
| **WHI** |  |
| Participants, n | 240 |
| Age, year | 68.0 ± 6.2 |
| BMI, kg/m^2^ | 29.1 ± 5.4 |
| Coffee, cups/d | 0 |
| Physical activity, MET-h/wk | 11.0 ± 13.4 |
| AHEI score | 54.7 ± 10.3 |
| Total energy intake, kcal/d | 1509 ± 709 |
| Alcohol, g/d | 3.7 ± 14.4 |
| Current smokers, % | 4.2 |
| Sugar-sweetened beverages, serving/d | 0.92 ± 1.05 |
| Genetic risk score | 70.7 ± 5.7 |

Plus-minus values are means ± SD.

**Table S3. Genetic associations with differences in BMI and prevalent obesity, according to coffee consumption in the HPFS, NHS and WHI (excluding non-coffee consumers)**

| Cohort | Study participants number | Coffee consumption | | | P for interaction |
| --- | --- | --- | --- | --- | --- |
|  |  | <1 cup/d | 1-3 cups/d | >3 cups/d |  |
| **BMI, kg/m^2^** |  |  |  |  |  |
| HPFS | 4322 | 0.81 ± 0.32 | 0.81 ± 0.25 | 0.32 ± 0.30 | 0.017 |
| NHS | 8934 | 1.75 ± 0.22 | 1.07 ± 0.13 | 1.13 ± 0.16 | 0.041 |
| WHI | 5408 | 1.80 ± 0.35 | 1.02 ± 0.17 | 0.96 ± 0.25 | 0.035 |
| Pooled* | 18664 | 1.52 ± 0.16 | 1.02 ± 0.10 | 0.95 ± 0.12 | <0.001 |
| **Obesity** |  |  |  |  |  |
| HPFS | 4322 | 2.17 (1.61-2.93) | 1.47 (1.16-1.85) | 1.31 (1.03-1.66) | 0.012 |
| NHS | 8934 | 1.98 (1.63-2.42) | 1.52 (1.34-1.73) | 1.50 (1.29-1.75) | 0.050 |
| WHI | 5408 | 1.56 (1.21-2.00) | 1.46 (1.28-1.66) | 1.40 (1.15-1.70) | 0.286 |
| Pooled* | 18664 | 1.88 (1.64-2.16) | 1.49 (1.37-1.62) | 1.43 (1.28-1.59) | 0.003 |

Data are β coefficients ± SE for differences in BMI (kg/m^2^) per increment of 10 risk alleles, or ORs (95% CIs) for prevalent obesity per increment of 10 risk alleles.

In the HPFS and NHS, data were derived from the repeated-measures analysis in men (3 measures from 1986 to 1998) and women (3 measures from 1986 to 1998); in the WHI, data were derived from the repeated-measures in women (2 measures from 1993 to 2003).

Data were adjusted for age, genotyping source, physical activity (<3, 3-8.9, 9-17.9, 18-26.9, ≥27 MET-h/wk), AHEI score (quintiles), total energy intake (quintiles), smoking status (never, former, current), sugar-sweetened beverages consumption (quintiles), and alcohol consumption (0, 0.1-4.9, 5-9.9, 10-14.9, ≥15 g/d).

*Results for the three cohorts were pooled by means of inverse-variance-weighted random effects meta-analysis (if P <0.05 for heterogeneity) or fixed effects meta-analysis (if P ≥0.05 for heterogeneity).

**Table S4. Genetic associations with differences in BMI and prevalent obesity, according to coffee consumption in the HPFS, NHS and WHI (only in non-smokers)**

| Cohort | Study participants number | Coffee consumption | | | P for interaction |
| --- | --- | --- | --- | --- | --- |
|  |  | <1 cup/d | 1-3 cups/d | >3 cups/d |  |
| **BMI, kg/m^2^** |  |  |  |  |  |
| HPFS | 2376 | 0.72 ± 0.31 | 1.15 ± 0.39 | 0.67 ± 0.60 | 0.800 |
| NHS | 4427 | 1.48 ± 0.24 | 1.09 ± 0.20 | 0.94 ± 0.25 | 0.110 |
| WHI | 2883 | 1.54 ± 0.36 | 1.33 ± 0.23 | 0.53 ± 0.40 | 0.052 |
| Pooled* | 9686 | 1.27 ± 0.17 | 1.19 ± 0.14 | 0.81 ± 0.20 | 0.032 |
| **Obesity** |  |  |  |  |  |
| HPFS | 2376 | 1.91 (1.33-2.74) | 1.53 (1.03-2.28) | 1.31 (0.79-2.17) | 0.199 |
| NHS | 4427 | 1.74 (1.40-2.15) | 1.46 (1.20-1.78) | 1.51 (1.16-1.97) | 0.268 |
| WHI | 2883 | 1.70 (1.30-2.23) | 1.62 (1.35-1.94) | 1.30 (0.96-1.76) | 0.117 |
| Pooled* | 9686 | 1.75 (1.51-2.04) | 1.54 (1.36-1.75) | 1.40 (1.16-1.69) | 0.023 |

Data are β coefficients ± SE for differences in BMI (kg/m^2^) per increment of 10 risk alleles, or ORs (95% CIs) for prevalent obesity per increment of 10 risk alleles.

In the HPFS and NHS, data were derived from the repeated-measures analysis in men (3 measures from 1986 to 1998) and women (3 measures from 1986 to 1998); in the WHI, data were derived from the repeated-measures in women (2 measures from 1993 to 2003).

Data were adjusted for age, genotyping source, physical activity (<3, 3-8.9, 9-17.9, 18-26.9, ≥27 MET-h/wk), AHEI score (quintiles), total energy intake (quintiles), sugar-sweetened beverages consumption (quintiles), and alcohol consumption (0, 0.1-4.9, 5-9.9, 10-14.9, ≥15 g/d).

*Results for the three cohorts were pooled by means of inverse-variance-weighted random effects meta-analysis (if P <0.05 for heterogeneity) or fixed effects meta-analysis (if P ≥0.05 for heterogeneity).

**Table S5. Baseline characteristics of decaffeinated coffee consumers and caffeinated coffee consumers in the HPFS and NHS**

| Characteristic | Decaffeinated coffee consumer | Caffeinated coffee consumer |
| --- | --- | --- |
| **HPFS** |  |  |
| Participants, n | 2699 | 3615 |
| Age, year | 55.0 ± 8.6 | 54.4 ± 8.6 |
| BMI, kg/m^2^ | 25.4 ± 4.6 | 25.4 ± 4.7 |
| Coffee, cups/d | 2.46 ± 1.86 | 2.64 ± 1.79 |
| Physical activity, MET-h/wk | 20.2 ± 23.6 | 19.1 ± 24.4 |
| AHEI score | 54.3 ± 11.7 | 52.3 ± 11.7 |
| Total energy intake, kcal/d | 2028 ± 597 | 2054 ± 619 |
| Alcohol, g/d | 12.7 ± 15.5 | 14.0 ± 16.6 |
| Current smoker, % | 7.6 | 10.0 |
| Sugar-sweetened beverages, serving/d | 1.11 ± 0.96 | 1.14 ± 1.00 |
| **NHS** |  |  |
| Participants, n | 5938 | 7709 |
| Age, year | 54.1 ± 6.6 | 54.1 ± 6.5 |
| BMI, kg/m^2^ | 25.4 ± 4.8 | 25.7 ± 4.9 |
| Coffee, cups/d | 2.65 ± 1.67 | 2.84 ± 1.61 |
| Physical activity, MET-h/wk | 14.7 ± 19.6 | 13.7 ± 18.4 |
| AHEI score | 50.4 ± 10.4 | 49.4 ± 10.3 |
| Total energy intake, kcal/d | 1783 ± 488 | 1782 ± 493 |
| Alcohol, g/d | 6.6 ± 9.9 | 7.4 ± 10.9 |
| Current smoker, % | 15.4 | 19.2 |
| Sugar-sweetened beverages, serving/d | 0.99 ± 0.55 | 0.98 ± 0.83 |

Plus-minus values are means ± SD.

Baseline data were from 41,213 men in the HPFS (1986) and 79,328 women in the NHS (1986). Participants were initially healthy men and women for whom baseline coffee consumption data were available. Decaffeinated coffee consumers and caffeinated coffee consumers overlapped.

**Table S6. Differences in BMI per increment of 10 risk alleles, according to consumptions of decaffeinated coffee and caffeinated coffee in the HPFS and NHS**

| Analysis | Coffee consumption | | | P for interaction |
| --- | --- | --- | --- | --- |
|  | <1 cup/d | 1-3 cups/d | >3 cups/d |  |
| **Decaffeinated coffee** |  |  |  |  |
| HPFS | 0.70 ± 0.17 | 0.68 ± 0.37 | -0.11 ± 0.86 | 0.280 |
| NHS | 1.34 ± 0.10 | 0.85 ± 0.20 | 0.83 ± 0.38 | 0.018 |
| Pooled* | 1.17 ± 0.09 | 0.82 ± 0.18 | 0.68 ± 0.35 | 0.010 |
| **Caffeinated coffee** |  |  |  |  |
| HPFS | 0.77 ± 0.21 | 0.93 ± 0.27 | -0.11 ± 0.37 | 0.058 |
| NHS | 1.32 ± 0.13 | 1.04 ± 0.14 | 1.32 ± 0.22 | 0.760 |
| Pooled* | 1.16 ± 0.11 | 1.02 ± 0.13 | 0.94 ± 0.19 | 0.212 |

Plus-minus values are β coefficients ± SE. In the HPFS and NHS, data were derived from the repeated-measures analysis in men (3 measures from 1986 to 1998) and women (3 measures from 1986 to 1998).

Data were adjusted for age, genotyping source, physical activity (<3, 3-8.9, 9-17.9, 18-26.9, ≥27 MET-h/wk), AHEI score (quintiles), total energy intake (quintiles), smoking status (never, former, current), sugar-sweetened beverages consumption (quintiles), and alcohol consumption (0, 0.1-4.9, 5.0-9.9, 10.0-14.9, ≥15 g/d); decaffeinated and caffeinated coffee were adjusted for each other.

*Results for the two studies were pooled by means of inverse-variance-weighted random effects meta-analysis (if P <0.05 for heterogeneity) or fixed effects meta-analysis (if P ≥0.05 for heterogeneity).

**Table S7. Comparison of baseline characteristics for study participants and the other participants in the HPFS and NHS**

| Characteristic | Study participants | Other participants | P |
| --- | --- | --- | --- |
| **HPFS** |  |  |  |
| Participants, n | 5116 | 36097 | - |
| Age, year | 54.4 ± 8.7 | 52.7 ± 9.6 | <0.001 |
| BMI, kg/m^2^ | 25.4 ± 4.7 | 24.9 ± 5.0 | <0.001 |
| Whites, % | 100.0 | 94.5 | <0.001 |
| Coffee, cups/d | 2.09 ± 1.87 | 1.92 ± 1.80 | <0.001 |
| Physical activity, MET-h/wk | 19.5 ± 24.8 | 21.7 ± 30.7 | <0.001 |
| AHEI score | 52.6 ± 11.8 | 52.5 ± 11.5 | 0.42 |
| Total energy intake, kcal/d | 2037 ± 617 | 1991 ± 622 | <0.001 |
| Alcohol, g/d | 12.5 ± 16.3 | 11.2 ± 15.3 | <0.001 |
| Current smoker, % | 8.6 | 9.9 | <0.001 |
| Sugar-sweetened beverages, serving/d | 1.17 ± 1.07 | 1.24 ± 1.19 | <0.001 |
| **NHS** |  |  |  |
| Participants, n | 9841 | 69487 | - |
| Age, year | 54.1 ± 6.6 | 51.9 ± 7.2 | <0.001 |
| BMI, kg/m^2^ | 25.8 ± 5.0 | 25.2 ± 4.8 | <0.001 |
| Whites, % | 100.0 | 97.4 | <0.001 |
| Coffee, cups/d | 2.43 ± 1.73 | 2.43 ± 1.76 | 0.91 |
| Physical activity, MET-h/wk | 14.0 ± 18.3 | 14.2 ± 21.2 | 0.35 |
| AHEI score | 49.5 ± 10.5 | 48.4 ± 10.4 | <0.001 |
| Total energy intake, kcal/d | 1770 ± 499 | 1753 ± 510 | 0.002 |
| Alcohol, g/d | 6.8 ± 10.7 | 6.6 ± 10.7 | 0.060 |
| Current smoker, % | 17.4 | 22.3 | <0.001 |
| Sugar-sweetened beverages, serving/d | 1.00 ± 0.87 | 0.98 ± 0.90 | 0.19 |

Plus-minus values are means ± SD.

Baseline data were from 41,213 men in the HPFS (1986) and 79,328 women in the NHS (1986). Participants were initially healthy men and women for whom baseline coffee consumption data were available.

**Figure S1. Distribution of the genetic risk score in the HPFS, NHS and WHI**


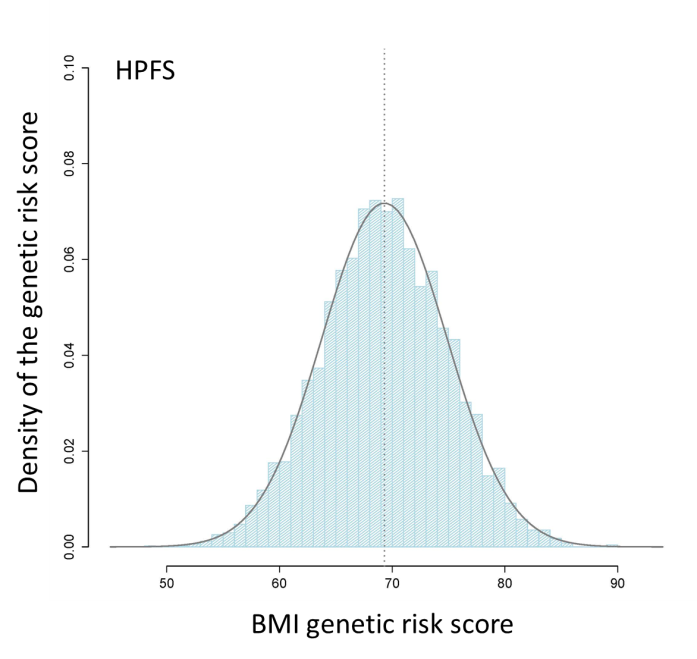


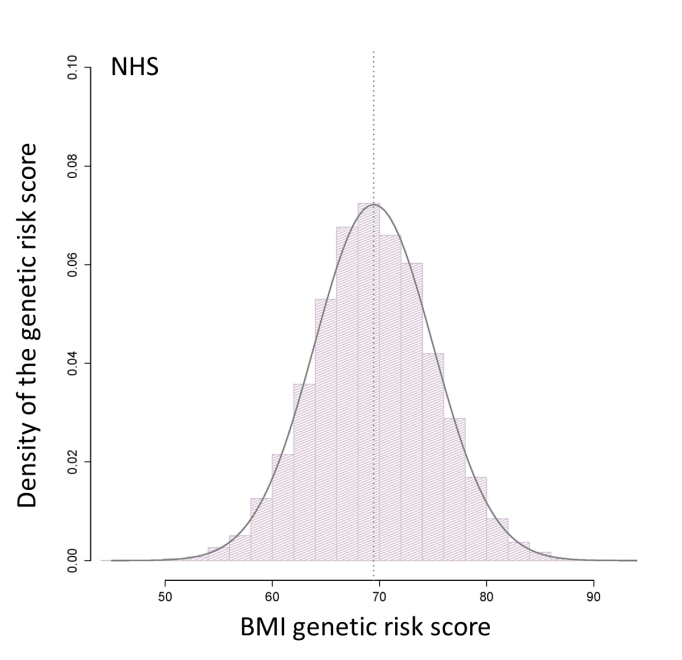


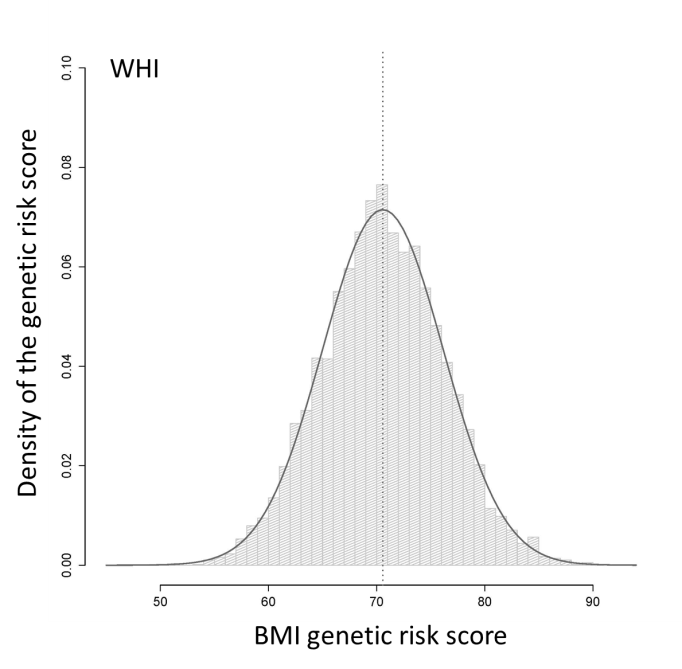


**Figure S2. Differences in BMI per increment of 1 cup/d decaffeinated coffee and caffeinated coffee consumption, according to tertiles of the genetic risk score in the HPFS and NHS**

Data are β coefficients ± SE.

Data description and adjustment are the same as shown in Supplementary Table 2.


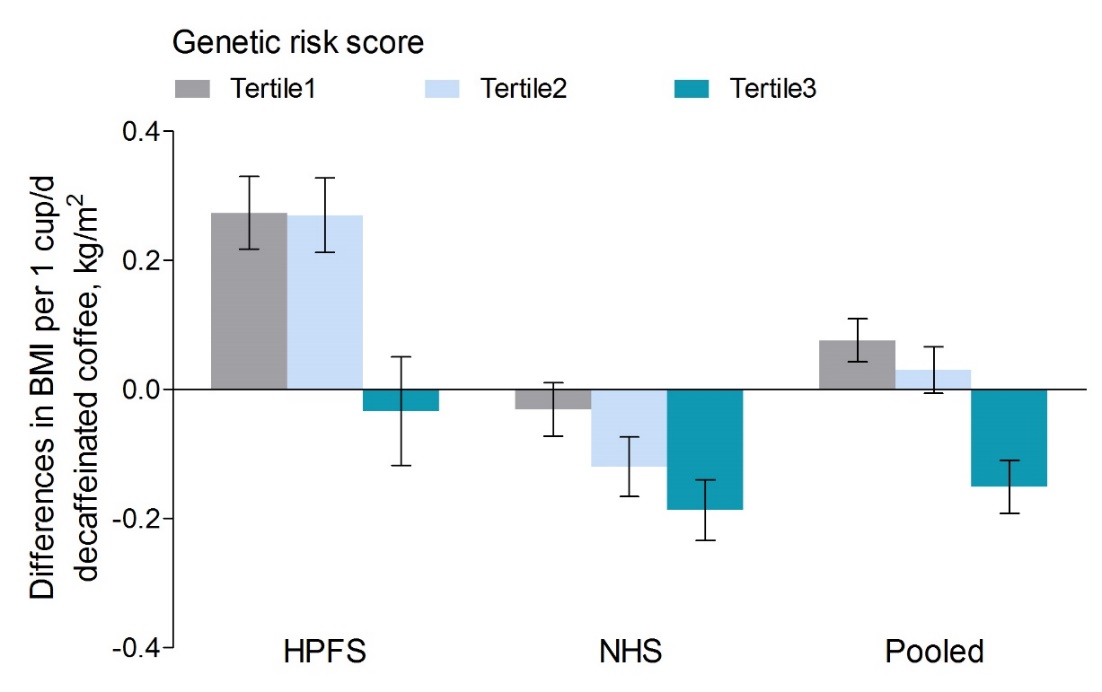


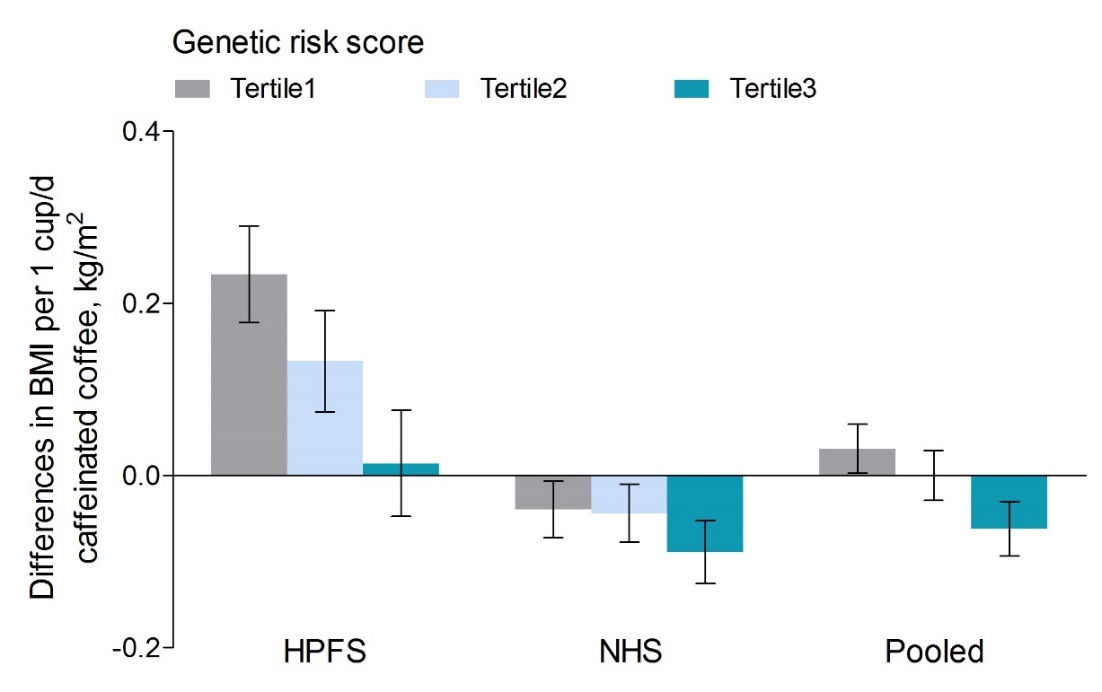

Supplement: Additional file 1: Table S1. — Characteristics of 77 SNPs for BMI in HPFS, NHS, and WHI. Table S2. Baseline characteristics of non-coffee consumers in HPFS, NHS, and WHI. Table S3. Genetic associations with differences in BMI and prevalent obesity, according to coffee consumption in HPFS, NHS, and WHI (excluding non-coffee consumers). Table S4. Genetic associations with differences in BMI and prevalent obesity, according to coffee consumption in HPFS, NHS and WHI (only in non-smokers). Table S5. Baseline characteristics of decaffeinated coffee consumers and caffeinated coffee consumers in HPFS and NHS. Table S6. Differences in BMI per increment of 10-risk alleles, according to consumption of decaffeinated coffee and caffeinated coffee in HPFS and NHS. Table S7. Comparison of baseline characteristics for study participants and the other participants in the HPFS and NHS. Figure S1. Distribution of the genetic risk score in the HPFS, NHS, and WHI. Figure S2. Differences in BMI per increment of 1 cup/day decaffeinated coffee and caffeinated coffee consumption, according to tertiles of the genetic risk score in HPFS and NHS. (DOCX 687 kb) [file 12916_2017_862_MOESM1_ESM.docx]
